# Novel parasitic chytrids infecting snow algae in an alpine snow ecosystem in Japan

**DOI:** 10.3389/fmicb.2023.1201230

**Published:** 2023-06-20

**Authors:** Hiroaki Nakanishi, Kensuke Seto, Nozomu Takeuchi, Maiko Kagami

**Affiliations:** ^1^Graduate School of Environment and Information Sciences, Yokohama National University, Yokohama, Kanagawa, Japan; ^2^Faculty of Environment and Information Sciences, Yokohama National University, Yokohama, Kanagawa, Japan; ^3^Department of Earth Sciences, Graduate School of Science, Chiba University, Chiba, Japan

**Keywords:** snow algae, fungi, chytrid, host–parasite interactions, snowpacks, polar and alpine ecosystems

## Abstract

**Introduction:**

Microbial communities are important components of glacier and snowpack ecosystems that influence biogeochemical cycles and snow/ice melt. Recent environmental DNA surveys have revealed that chytrids dominate the fungal communities in polar and alpine snowpacks. These could be parasitic chytrids that infect snow algae as observed microscopically. However, the diversity and phylogenetic position of parasitic chytrids has not been identified due to difficulties in establishing their culture and subsequent DNA sequencing. In this study, we aimed to identify the phylogenetic positions of chytrids infecting the snow algae, *Chloromonas spp.*, bloomed on snowpacks in Japan.

**Methods:**

By linking a microscopically picked single fungal sporangium on a snow algal cell to a subsequent sequence of ribosomal marker genes, we identified three novel lineages with distinct morphologies.

**Results:**

All the three lineages belonged to Mesochytriales, located within “Snow Clade 1”, a novel clade consisting of uncultured chytrids from snow-covered environments worldwide. Additionally, putative resting spores of chytrids attached to snow algal cells were observed.

**Discussion:**

This suggests that chytrids may survive as resting stage in soil after snowmelt. Our study highlights the potential importance of parasitic chytrids that infect snow algal communities.

## 1. Introduction

Seasonal snowpacks are inhabited by diverse organisms of various taxonomic groups, including photosynthetic primary producers (snow algae and cyanobacteria) (Takeuchi, 2001; Hoham and Remias, 2020) and heterotrophic microorganisms (bacteria, fungi, and micro invertebrates) (Yakimovich et al., 2020; Fiołka et al., 2021; Kobayashi et al., 2023). During snowmelt, snow algae grow on snow surfaces, which covers the surface with green or red color (Hoham and Duval, 2001). Colored snow is typically dominated by algae belonging to the phylum Chlorophyta (Procházková et al., 2019; Hoham and Remias, 2020; Nakashima et al., 2021; Ono et al., 2021). Snow algae also accelerate snowmelt, as they can reduce the albedo of snow surface, resulting in increased absorption of solar radiation by snow (Lutz et al., 2016; Ganey et al., 2017; Hotaling et al., 2021). Several taxonomic studies have been performed on snow algae to elucidate their ecology (Matsuzaki et al., 2015, 2018, 2019; Segawa et al., 2018; Procházková et al., 2019). Some bacteria and fungi have symbiotic or parasitic relationships with snow algae, and they may affect snow algal population (Terashima et al., 2017). Recently, some studies have attempted to characterize the snow algae–bacteria relationships using 16S rRNA gene amplicon sequencing and co-cultivation with snow algae (Terashima et al., 2017; Krug et al., 2020). However, few studies have focused on the snow algae–fungus relationship, and the taxonomic knowledge of this relationship is particularly poor.

Recent environmental DNA analyses have revealed that diverse fungi inhabit alpine snowpacks worldwide and chytrids often dominate fungal communities (Freeman et al., 2009; Schmidt et al., 2012; Naff et al., 2013; Brown et al., 2015; Yakimovich et al., 2020). Novel clades composed mainly of environmental DNA sequences from snow-covered regions were identified in Chytridiomycota (Naff et al., 2013). However, as these chytrid sequences were directly obtained from soil and snow samples and not from cultures, their morphology, life cycles, and ecology remain unknown.

Microscopic observations have shown that chytrids infect algae in alpine snowpacks and glaciers. Kol (1942) reported that a chytrid, morphologically identified as *Rhizophidium sphaerocarpum*, infects the glacier alga *Ancylonema nordenskioldii* on a glacier in Alaska. Additionally, chytrids infecting the snow alga *Sanguina nivaloides* (formerly known as *Chlamydomonas nivalis*) have been observed in polar and alpine snowpacks (Kobayashi and Okubo, 1954; Kol, 1968; Yakimovich et al., 2020; Fiołka et al., 2021; Kobayashi et al., 2023). As observed in lakes, these chytrids may suppress algal populations and affect trophic dynamics in snow ecosystems (Kagami et al., 2007; Frenken et al., 2017). However, as they have not yet been cultured, their diversity and phylogenetic positions have not been determined.

This study aimed to determine the phylogenetic position of parasitic chytrids that infect snow algae. We used single-spore PCR to sequence uncultured chytrids in the bloom of snow alga *Chloromonas* spp. in alpine snowpacks in Japan. This method enabled us to directly link microscopic observations to DNA sequencing without culturing (Ishida et al., 2015; Kagami et al., 2021; Van den Wyngaert et al., 2022). This study provides the first phylogenetic evidence of parasitic chytrids infecting snow algae in alpine regions.

## 2. Materials and methods

### 2.1. Study site

**Figure 1:**
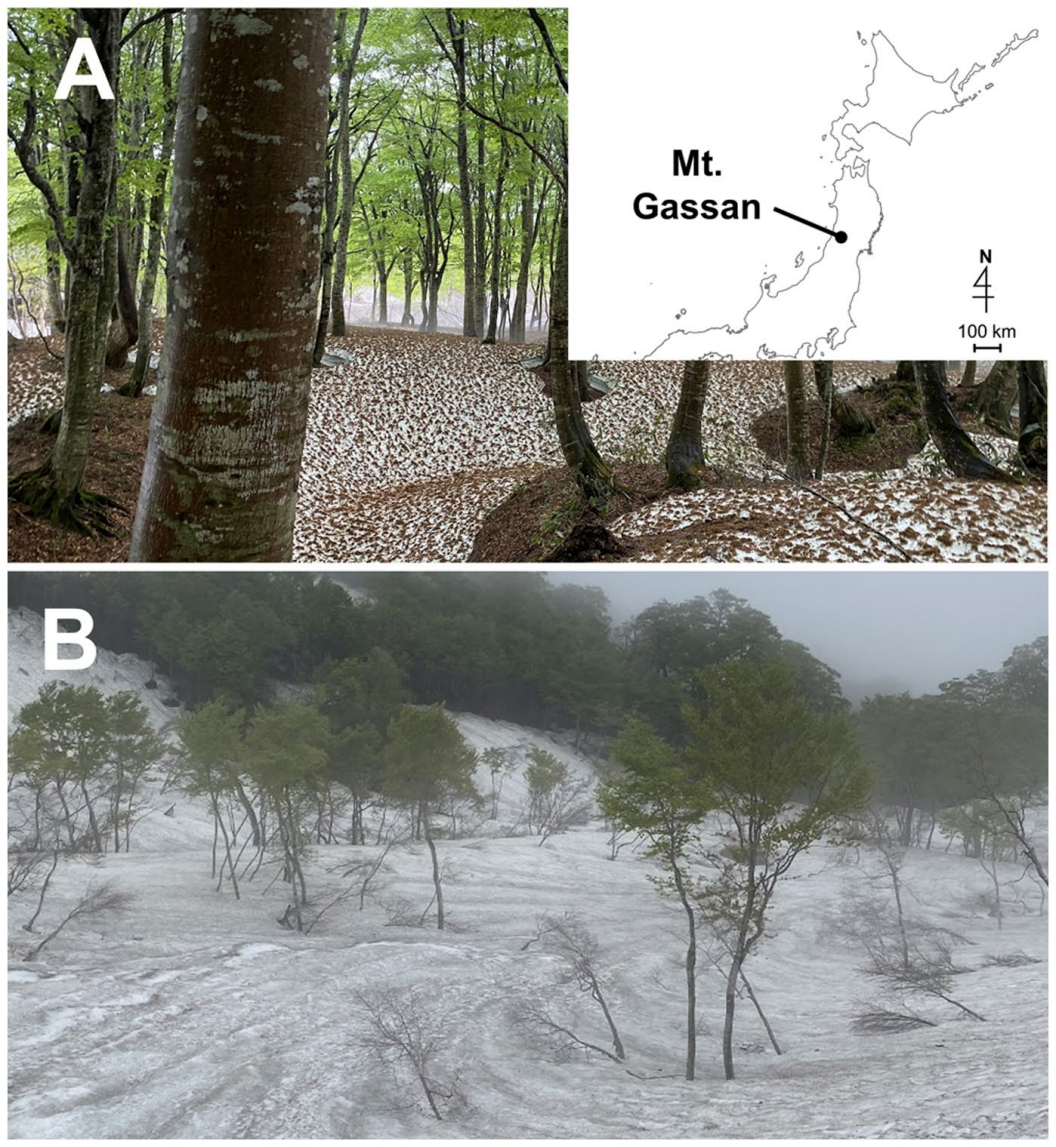
Photographs of the landscape of the sampling sites on Mt. Gassan in Japan. **(A)**: Site A (770  m a. s. l.). The map shows the location of Mt. Gassan. **(B)**: Site B (1150  m a. s. l.).

**Figure 2:**
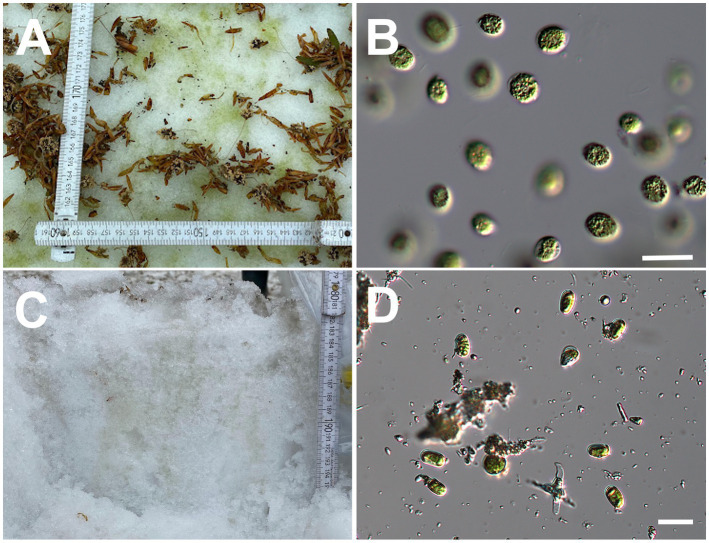
**(A)**: Green snow at Site A and **(B)**: micrograph of the green snow at Site A. **(C)**: Vertical section of green snow at Site B and **(D)**: micrograph of the green snow at Site B. All scale bars: 20  μm.

The field study was performed on Mt. Gassan, Yamagata prefecture in Japan at two sites, Site A (38° 29′ N, 140° 00′ E 770 m above sea level (a. s. l.); Figure 1A) and Site B (38° 31′ N, 140° 00′ E 1,150 m a. s. l.; Figure 1B). A large amount of snow accumulates in Mount Gassan every winter (Kariya, 2005). At Sites A and B, green snow appeared from late April to mid-June (Figures 2A,C). The vegetation at the study site is dominated by mountain broad-leaved deciduous trees, including *Fagus crenata* shown in Figure 1A, up to an elevation of 1,500 m a. s. l.

### 2.2. Sample collection

Samples were collected from green snow that appeared in May 2021 and May 2022 at the two sites (Figures 2A,C). The 2021 samples were collected in 15 mL plastic tubes, stored in a box filled with snow, and transported to a laboratory. Some tubes were stored at −80°C for DNA analysis in a deep freezer (MDF-C8V1-PJ, Panasonic, Japan). The remaining tubes were incubated at 3°C under dim light 20 μmol photons m^−2^ s^−1^ and 12:12 h light:dark cycle in an incubator (SLC-25A, Mitsubishi Electric Engineering, Japan). The incubated samples were observed under a light microscope (IX71, Olympus, Japan) every few days to identify parasitic chytrids infecting *Chloromonas* spp.

### 2.3. Microscopic observation

**Figure 3:**
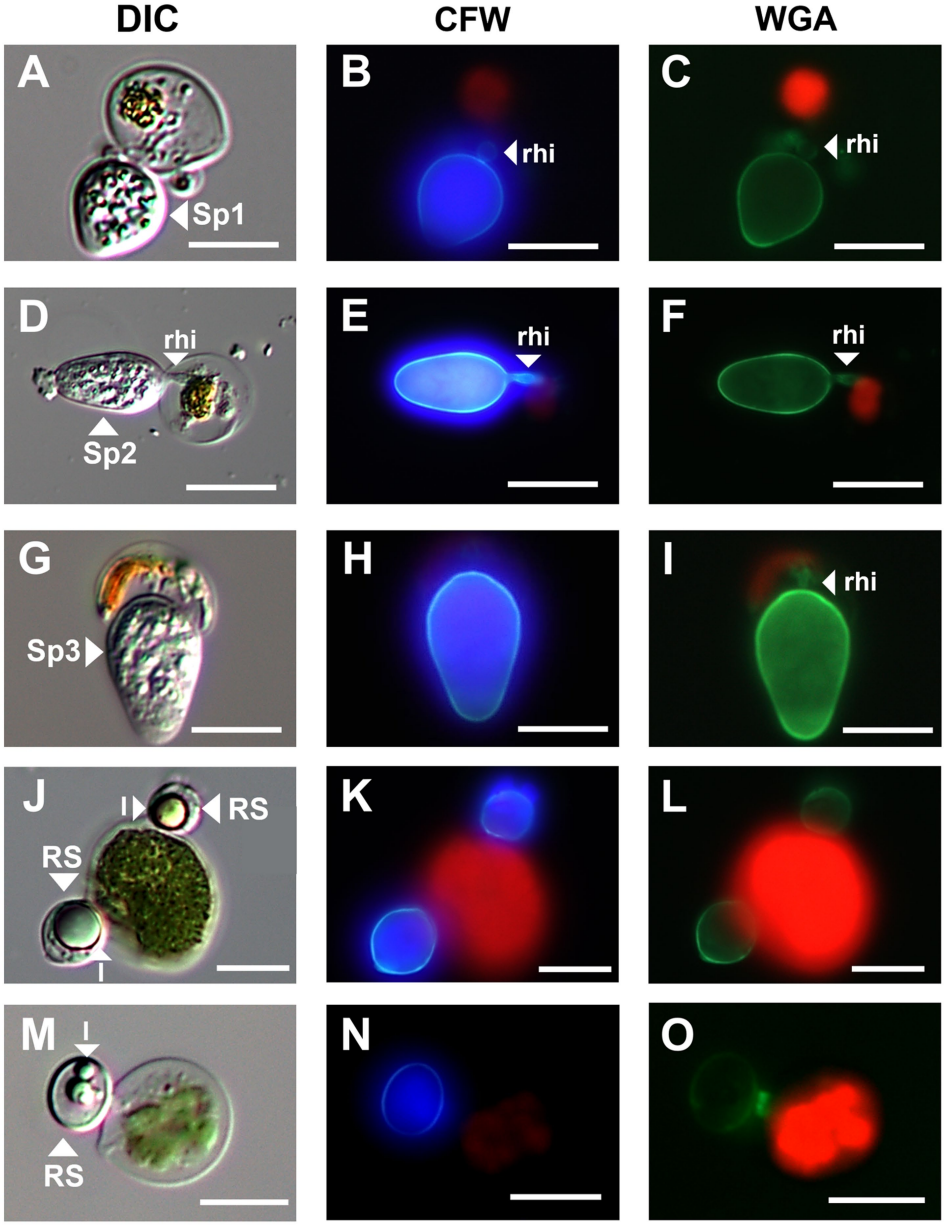
Micrographs of three sporangium type and resting spore of parasitic chytrids infecting *Chloromonas* spp. **(A–C)**; Sp1, **(D–F)**; Sp2, **(G–I)**; Sp3, **(J–O)**; resting spores. All scale bars are 10 μm. DIC, differential interference contrast; CFW, under blue fluorescent excitation after CFW staining; WGA, under green fluorescent excitation after WGA staining; rhi, rhizoids; l, lipid globules; RS, resting spore.

Many snow algal cells of *Chloromonas* spp. were present in the green snow at both the sites (Figures 2B,D). After calcofluor white fluorescent (CFW) –wheat germ agglutinin (WGA) double staining (Klawonn et al., 2023), the samples were observed under a microscope to identify parasitic chytrids infecting *Chloromonas* spp. Green snow samples were aliquoted into 1 mL Eppendorf tubes, and 5 μg mL^−1^ of CFW (Fluorescent Brightener 28, Sigma Aldrich, United States) and 5 μg mL^−1^ of WGA (Wheat Germ Agglutinin, Alexa Fluor^™^ 488 conjugate, Thermo Fisher Scientific, United States) were added. CFW and WGA can be used simultaneously because they have different binding target structures and absorb at different wavelengths, making the detection of chitin in fungal cell walls reliable (Klawonn et al., 2023). Photographs were taken using a digital camera (Advancam 305 color, Carl Zeiss, Germany) at differential interference contrast, blue fluorescence excitation (CFW), and green fluorescence excitation (WGA) on a fluorescence microscope (AXIO Imager. M2, Carl Zeiss, Germany). Based on the micrographs, the chytrids were morphologically typed (Figure 3). Chytrids with thick cell walls that accumulated single or multiple intracellular lipid globules were classified as the resting spore (Seto et al., 2017). To calculate the prevalence of chytrid infection in algal cells, we counted the number of algal cells with and without chytrids in six samples collected at Site A in May 2021. More than 200 algal cells were counted in each sample. The prevalence of infection was calculated as the number of infected algal cells divided by the total number of algal cells counted.

### 2.4. Single-spore PCR

#### 2.4.1. Microscopic observation and isolation of parasitic chytrids

The cryopreserved and incubated samples were used for single-cell isolation. The cryopreserved samples were thawed at room temperature (approximately 20°C) before analysis. To stain the fungal cell wall, 5 μL of CFW was added to 1 mL of the samples. Chytrids infecting snow algae *Chloromonas* spp. were observed under an inverted fluorescence microscope (IX71, Olympus, Japan) and photographed using a digital camera (DP21, Olympus, Japan). The chytrid and host cells were isolated using a capillary pipette at room temperature. The isolated cells were washed three times with autoclaved deionized water to remove contamination of other fungi, and the cells were individually transferred into 200 μL PCR tubes with a small amount of deionized water.

#### 2.4.2. DNA extraction

DNA extraction was performed using the HotSHOT method (Truett et al., 2000). PCR tubes containing isolated cells were filled with 5 μL of alkaline lysis buffer (20 mM EDTA and 0.2 mM NaOH; pH 12) and heat-treated in a thermal cycler (MiniAmp Plus, Thermo Fisher Scientific, United States) at 96°C for 10 min and 5°C for 5 min. Subsequently, 5 μL of neutralization buffer (40 mM Tris hydrochloride, pH 5) was added and stored at −80°C.

#### 2.4.3. PCR amplification and sequencing

The extracted DNA was subjected to PCR amplification of the ribosomal RNA gene (rDNA) region in a thermal cycler using several fungus-specific primer sets (Table 1) and the DNA polymerase KOD FX Neo (TOYOBO, Japan). PCR was performed in 10 μL volume composed of 1.3 μL of sterile ultrapure water, 5 μL of 2× PCR Buffer for KOD FX Neo, 2 μL of dNTPs (2 mM), 0.2 μL of each primer (10 μM), 0.2 μL of KOD FX Neo, 0.1 μL of bovine serum albumin (50 mg mL^−1^, Wako, Japan), and 1 μL of extracted DNA. The thermal cycle for PCR was (1) 95°C for 5 min, (2) 12 cycles of denaturation at 95°C for 30 s, annealing at 54–48°C for 30 s (decrease of 0.5°C per cycle), extension at 68°C for 4 min, and (3) 23 cycles of 95°C for 30 s, 48°C for 30 s, and 68°C for 4 min. The PCR products were purified using ExoSAP-IT (Affymetrix, United States). Sequencing was performed using the commercial service of Eurofins Genomics in Japan with the sequencing primers listed in Table 2. Differences in the PCR success rate in isolated chytrids at sporangial stage and at resting spore stage were assessed by Fisher’s exact probability test. The statistical analysis was performed using R software (version 4.2.1; R Core Team, 2022).^1^

**Table 1:** List of primers used for PCR amplification of rDNA region of chytrids.

Genomic target	Name of the primer	Primer sequence (5′-3′)	References
18S rDNA	NS1short	CAGTAGTCATATGCTTGTC	Wurzbacher et al. (2019)
	AU4v2	GCCTCACTAAGCCATTC	Lazarus and James (2015)
ITS1-5.8S-ITS2	ITS1-F	CTTGGTCATTTAGAGGAAGTAA	Gardes and Bruns (1993)
	5.8S	CGCTGCGTTCTTCATCG	Vilgalys and Hester (1990)
	ITS4	TCCTCCGCTTATTGATATGC	White et al. (1990)
28S rDNA	LR0R	ACCCGCTGAACTTAAGC	Vilgalys and Hester (1990)
	LR5	TCCTGAGGGAAACTTCG	Vilgalys and Hester (1990)

**Table 2:** List of primers used for sanger sequencing, which Snow1-F and Snow2-R are primers newly applied in this study.

Genomic target	Name of the primer	Primer sequence (5′-3′)	References
18S rDNA	NS1short	CAGTAGTCATATGCTTGTC	Wurzbacher et al. (2019)
	Snow1-F	TTTTCGGAACCGAGGTAATG	This paper
	Snow2-R	GGTTCCGAAAACCAACAGAA	This paper
	NS4	CTTCCGTCAATTCCTTTAAG	White et al. (1990)
	NS8z	TCCGCAGGTTCACCTACG	O’Donnell et al. (1998)
	AU4v2	GCCTCACTAAGCCATTC	Lazarus and James (2015)
ITS1-5.8S-ITS2	ITS1-F	CTTGGTCATTTAGAGGAAGTAA	Gardes and Bruns (1993)
	5.8S	CGCTGCGTTCTTCATCG	Vilgalys and Hester (1990)
	ITS4	TCCTCCGCTTATTGATATGC	White et al. (1990)
28S rDNA	LR0R	ACCCGCTGAACTTAAGC	Vilgalys and Hester (1990)
	LR5	TCCTGAGGGAAACTTCG	Vilgalys and Hester (1990)

### 2.5. Molecular phylogenetic analysis

The sequences obtained were assembled using Chromas Pro ver. 2.1.10 (Technelysium, Australia). The assembled sequences of 18S, ITS, and 28S rDNA were deposited under the accession numbers LC761280–LC761291. BLAST searches performed using the rDNA sequences obtained indicated that all isolates belonged to the order Mesochytriales of Chytridiomycota. Therefore, we performed phylogenetic analysis of 18S rDNA using the new sequences obtained in this study because this marker region is the richest in reference sequences for Mesochytriales. For molecular phylogenetic analysis, a dataset of 18S rDNA sequences from Mesochytriales, Gromochytriales, and Polyphagales was used (Table 3). Lobulomycetales were included as outgroups. Sequences were aligned using MAFFT (Katoh et al., 2019) with default parameters. Ambiguously aligned sequences were trimmed using trimAl (Capella-Gutiérrez et al., 2009). Molecular phylogenetic analysis using the maximum likelihood method was performed using the IQ-TREE online server (Trifinopoulos et al., 2016).

**Table 3:** List of known species and environmental DNA sequences belonging to Mesochytriales, Gromochytriales, Polyphagales, and Lobulomycetales used in the molecular phylogenetic tree (Figure 4).

Name	GenBank accession no. (18S rDNA)	Habitat/Geographic location	Charactarization/Season	References
*Mesochytrium penetrans* CALU x-10	FJ804149	Lake water, Finland	Parasitic on green alga *Chlorococcum minutum*	Karpov et al. (2010)
*Gromochytrium mamkaevae* CALU x-51	KF586842	Ditch near town Kirovsk, Russia	Parasitic on yellow-green alga *Tribonama gayanum*	Karpov et al. (2014)
*Apiochytrium granulosporium* x-124	MK179157	Pond water, Russia	Parasitic on yellow-green alga *Tribonama gayanum*	Karpov et al. (2019)
*Lobulomyces angularis* JEL45	AF164253	Sphagnum from acidic lake, United States	Saprophytic or parasitic	Simmons et al. (2009)
*Clydaea vesicula* JEL0476	MT730721	Soil under Eucalyptus trees, United States	Saprophytic or parasitic	Simmons et al. (2009)
*Maunachytrium kenaense* AF021	EF432822	Alpine barren soil, United States	Saprophytic or parasitic	Simmons et al. (2009)
*Polyphagus parasiticus* Pp	KX449337	Pond water, Russia	Parasitic on yellow-green alga *Tribonama gayanum*	Karpov et al. (2016)
*Endocoenobium endorinae* SVdW-EUD1	MG605053	Lake Stechlin, Germany	Parasitic on Colonial Volvocacean Algae	Van den Wyngaert et al. (2018)
uncultured WS 10-E02, WS 10-E15	AJ867629, AJ867631	Lake Joeri XIII inflow of melt water, Switzerland	-	Unpublished data
Uncultured Spring_37	JX069054	River site, Southern Alberta, Canada	Spring	Thomas et al. (2012)
Uncultured T2P1AeB05, T2P1AeF04, T3P1AeC03, T5P2AeC07	GQ995415, GQ995412, GQ995413, GQ995414	High-elevation soil not far from ice and snow	July–October	Freeman et al. (2009)
Uncultured T31a_23, T31b_01	KC561971, KC561972	Rocky Mountain talus snow, Colorado, United States	July–August	Naff et al. (2013)
Uncultured E109_01C	KC561936	High mountain snow, Nepal	October	Naff et al. (2013)
Uncultured R11a_04	KC561955	Rocky Mountain talus snow, Colorado, United States	July–August	Naff et al. (2013)
Uncultured Clones from a lake in China	JX426910	Freshwater lake, China	-	Unpublished data
Uncultured PFF5SP2005, PFD6SP2005, PFA12SP2005	EU162641, EU162637, EU162643	Oligo-mesotrophic mountain Lake Pavin, France	May–June	Lefèvre et al. (2008)
Uncultured Pa2007C10	JQ689425	Oligo-mesotrophic mountain Lake Pavin, France	April	Jobard et al. (2012)
Uncultured Kili_01H_N5	KX771763	Glacier ice on Mt. Kilimanjaro, Tanzania	January	Unpublished data
Uncultured kor_110904_17	FJ157331	Lake Koronia water, Greece	November	Genitsaris et al. (2009)
Uncultured NKS146	JX296576	Hyposaline soda lake Nakuru, Kenya	November	Luo et al. (2013)
Uncultured FV23_1H5	DQ310332	Super-sulfidic anoxic fjord water, Norway	May	Behnke et al. (2006)

## 3. Results

### 3.1. Observation of parasitic chytrids infecting snow algae

The green snow samples collected in this study revealed the presence of chytrids at the sporangial stage and putative resting spores attached to the surface of vegetative cells of *Chloromonas* spp. (Figure 3). Chytrids at the sporangial stage were further categorized into three types based on their shape (Figure 3). Sporangium type 1 (Sp1) was egg shaped with a rounded swollen rhizoid (Figures 3A–C). Sporangium type 2 (Sp2) was ellipsoidal and had rod-shaped rhizoids (Figures 3D–F). Sporangium type 3 (Sp3) was immersed in host cells and had a rod-shaped rhizoid structure (Figures 3G–I). Putative resting spores of chytrids were clearly visible with thick cell walls and single or multiple large lipid globules (Figures 3J–O). Based on the observations of six green snow samples, the average prevalence of chytrid infection in the total vegetative algal cells observed was calculated to be 5.34%.

### 3.2. Single-spore PCR

In total, 79 chytrid cells were isolated from the snow algal hosts, of which 57 cells could be categorized into the three aforementioned sporangial stages: Sp1 (*n* = 11), Sp2 (*n* = 37), and Sp3 (*n* = 4). The other five sporangial cells could not be assigned to any sporangial type because they were immature small sporangia. We also isolated 22 cells from the putative resting spore stage of parasitic chytrids (Supplementary Table S1).

Single-spore PCR was performed using the 72 cells, and at least one of the three rDNA regions was successfully amplified in 33 cells (Supplementary Table S1). Chytrids at the resting spore stage had a significantly lower PCR success rate than those at the sporangial stage (value of *p* = 2.135 x 10^–5^, Supplementary Table S1).

Sanger sequencing was used to determine the partial or assembled sequences of rDNA regions of the 32 chytrid cells. Eleven cells were sequenced for their 18S rDNA regions. Four cells were Sp1, five were Sp2, and two were Sp3 (Supplementary Table S1). The sequences differed between the types but were identical within types.

### 3.3. Molecular phylogenetic analysis

**Figure 4:**
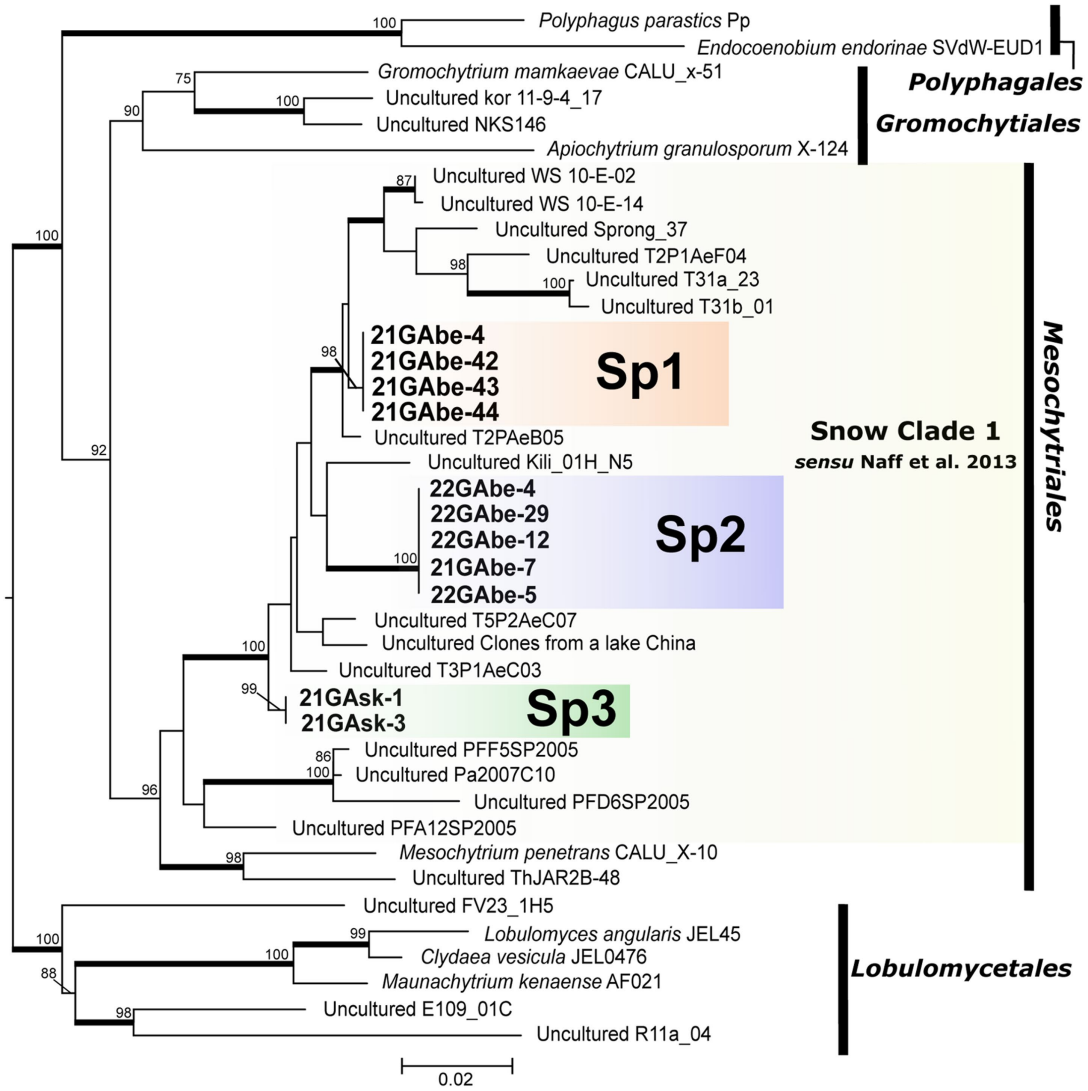
Maximum likelihood molecular phylogenetic tree showing the phylogenetic position of chytrid species belonging to Polyphagales, Gromochytriales, Mesochytriales, and Lobulomycetales using 18S rDNA. Lobulomycetales is the outgroup. Bold letters indicate samples detected in this study. Yellow shading indicates Snow Clade 1 proposed by Naff et al. (2013). Numbers above branches indicate values computed with UFBoot and only UFBoot ≥70% is indicated. Nodes supported by the SH-aLRT ≥95% are highlighted with a bold line. Genbank accession numbers for other sequences used in the phylogenetic analysis, as well as information on the source of isolation (culture strain) and detection point (environmental DNA sequence) are listed in Table 3.

Our molecular phylogenetic analysis showed that the 11 isolated cells of parasitic chytrids infecting snow algae clustered into three novel lineages (Sp1, Sp2, and Sp3) in Mesochytriales (Figure 4). They were included in Snow Clade 1 consisting of sequences from alpine snow-related environments located worldwide that are deeply divergent from the clade containing the known species *Mesochytrium penetrans*. Sp1 is a sister group of clades composed of environmental sequences obtained from lakes in Switzerland fed by abundant snow meltwater, river water from southwestern Alberta, Canada, and talus snows from the Rocky Mountains in Colorado, United States. Sp2 is closely related to the environmental sequences from glaciers on the summit of Mt. Kilimanjaro, Tanzania. Sp3 formed an independent clade that was divergent from the clade containing Sp1 and Sp2.

## 4. Discussion

This study successfully identified the phylogenetic position of three novel parasitic chytrids that infect snow algae in Japan. Mesochytriales, to which the three novel lineages belong, includes only one described species, *Mesochytrium penetrans*, which infected the green alga *Chlorococcum minutum*, isolated from a pond in Russia (Gromov et al., 2000; Karpov et al., 2010, 2014). Mesochytriales is one of the most understudied orders in the phylum Chytridiomycota and comprises many environmental DNA sequences (Karpov et al., 2014). Particularly, a group of environmental DNA sequences detected only in snow-related environments has been reported as Snow Clade 1 (Naff et al., 2013). It has been hypothesized that chytrids belonging to Snow Clade 1 spend at least a part of their life in snow and use either abundant snow algae or pollen as the nutritional resource (Naff et al., 2013). In this study, 11 chytrid cells infecting the snow algae *Chloromonas* spp. were found within Snow Clade 1 as independent lineages. This and earlier sequencing data suggest that chytrids infecting snow algae are phylogenetically diverse and present in various regions of the world. Furthermore, it can be assumed that the lineage composed of snow-derived environmental DNA sequences within Snow Clade 1 are parasitic chytrids infecting snow algae.

Our three species were morphologically distinguished from the previously described species, *Chytridium (Chy.) neochlamydococci*, parasitic chytrid of the snow alga *Chlamydomonas (Chla.) nivalis* (=*Sanguina nivaloides*) (Kobayashi and Okubo, 1954). *Chytridium neochlamydococci* formed epibiotic and citriform sporangia of 8–10 μm in length, and rhizoids with 2–3 branches from the base, which was originally found from the snow at Ozegahara Moor in Japan (Kobayashi and Okubo, 1954). Contrary to *Chy. neochlamydococci,* our Sp3 uniquely had sporangia partially embedded in the host cell. The Sp1 and Sp2 differed in other aspects; (i) the Sp1 had egg-shaped sporangia, (ii) the Sp2 had ellipsoidal and larger (10–15 μm in length), (iii) both our species had swollen or rod-shaped rhizoids without branches. The other described chytrid, *Chy.* f. *cryophile* (Kol, 1968), a parasite of *Chla. nivalis*, was not comparable due to the lack of morphological descriptions. Since the host of the chytrid was different from the hosts of our three lineages *Chloromonas* spp., they were probably different species.

Although parasitic chytrids generally reproduce under aquatic conditions, there are sequences in Snow Clade 1 that have been abundantly detected in soils after the disappearance of snow cover (Freeman et al., 2009). These sequences may have been derived from chytrids in their resting spore stage. In this study, a putative resting-spore stage of chytrids was identified in a snowpack. They were positive for CFW–WGA double staining and had typical structures of resting spores, thick cell walls, and large lipid globules accumulated within the cells, allowing them to survive in soils exposed to high temperatures and desiccation after snowmelt. Likewise, snow algae commonly encyst to form persistent resting spores and adhere to the soil upon melting of the snowpack (Hoham and Duval, 2001; Řezanka et al., 2008; Remias et al., 2010). The resting spores are believed to germinate at the snow-soil interface during the next snowmelt season, swim up to the snow surface, and reproduce (Hoham and Duval, 2001; Hoham and Remias, 2020). This strategy allows them to survive even at unsuitable reproduction times.

Based on these results, we hypothesized that parasitic chytrids infecting snow algae begin to grow after germination of the resting spores of chytrids during the snowmelt season, reproduce for a limited period, and then, rest in soil. Parasitic chytrids that infect algae in lakes are believed to form resting spores at the end of algal blooms, allowing them to survive in the absence of their hosts (Van Donk and Ringelberg, 1983). Germination of the resting spores of parasitic chytrids is also observed in several species within the genus *Synchytrium*, which infect cucurbitaceous plants. These resting spores germinate when they are exposed to high temperatures and moisture for more than a certain period of time (Raghavendra Rao and Pavgi, 1979). Further studies on parasitic chytrids infecting snow algae are needed to understand their life histories and influence on algal populations.

No phylogenetic information on the resting spores of chytrids was found in this study because single-spore PCR and sequencing were unsuccessful for all 22 cells. Resting spores of chytrids form a thick cell wall, and the dissolution of cell wall using the HotSHOT method may be insufficient. Additionally, as there is only one nucleus in resting spores, the amount of DNA is significantly lower than that in chytrids at the sporangial stage, which contain multiple nuclei. To unify the sporangial and resting spore stages in the same lineage, it is necessary to establish a two-member culture system with the host, and induce this in experiments. This will allow us to determine the consistent life histories of parasitic chytrids that infect snow algae. Furthermore, genome sequencing of cultured strains may provide genetic insights into the evolutionary history of chytrid adaptation to snow environments.

To the best of our knowledge, this study provides the first phylogenetic evidence of parasitic chytrids infecting snow algae in alpine regions. Identification using culture-independent single-spore PCR provided insights into the detailed diversity of these chytrids. However, we could not assess the impact of chytrids on the population dynamics of snow algae and snowpack ecosystems. In this study, the prevalence of chytrid infection in the vegetative cells of *Chloromona*s spp. in green snow samples was 5.34%. This low prevalence does not necessarily indicate a low impact of chytrids on algal dynamics. In lakes, chytrids significantly suppress algal populations, but prevalence of infection was not always high. There is often a time lag between the spread of chytrids infecting algae in the lake and peak algal bloom (Gsell et al., 2013). Spatial variation in the prevalence of infection could be significant, as reported for a glacier in which more glacial algal cells were infected by chytrids in water-filled holes on the glacier (cryoconite holes) than those on the ice surface (Kobayashi et al., 2023). In alpine snowpacks, the increased water content of snow may also spread chytrids that infect snow algae. Since the beginning of the 21st century, alpine snow cover has been declining globally due to climate change (Hock et al., 2019). Changes in snowfall level due to global warming have been reported in the high mountainous areas of Japan (Kawase et al., 2020). Therefore, in these areas, chytrids infecting snow algae may have increased prevalence and inhibit the albedo-decreasing effects of snow algae.

Our findings highlight the importance of parasitic chytrids that infect snow algae in various regions of the world. Further studies warranted to understand the impact of chytrid infection on snow algal dynamics and predict how their dynamics will be affected by climate change.

## Data availability statement

The datasets presented in this study can be found in online repositories. The names of the repository/repositories and accession number(s) can be found in the article/Supplementary material.

## Author contributions

HN, KS, and MK designed the study. HN, NT, and MK collected the samples. HN performed microscopic observation and single-spore PCR. HN and KS performed molecular phylogenetic analysis. HN wrote the manuscript with major input from KS, NT, and MK. All authors discussed the results and contributed to the final manuscript.

## Funding

This study was supported by a JSPS KAKENHI Grant-in-Aid for Scientific Research (22H03731, 20K21840, and 19H01143).

## Conflict of interest

The authors declare that the research was conducted in the absence of any commercial or financial relationships that could be construed as a potential conflict of interest.

## Publisher’s note

All claims expressed in this article are solely those of the authors and do not necessarily represent those of their affiliated organizations, or those of the publisher, the editors and the reviewers. Any product that may be evaluated in this article, or claim that may be made by its manufacturer, is not guaranteed or endorsed by the publisher.

## Footnotes

^1^ https://www.R-project.org/

## References

[ref1] Behnke A. Bunge J. Barger K. Breiner H.-W. Alla V. Stoeck T. (2006). Microeukaryote community patterns along an O2/H2S gradient in a supersulfidic anoxic fjord (Framvaren, Norway). Appl. Environ. Microbiol. 72, 3626–3636. doi: 10.1128/AEM.72.5.3626-3636.2006 16672511 PMC1472314

[ref2] Brown S. P. Olson B. J. S. C. Jumpponen A. (2015). Fungi and algae co-occur in snow: an issue of shared habitat or algal facilitation of heterotrophs? Arct. Antarct. Alp. Res. 47, 729–749. doi: 10.1657/AAAR0014-071

[ref3] Capella-Gutiérrez S. Silla-Martínez J. M. Gabaldón T. (2009). trimAl: a tool for automated alignment trimming in large-scale phylogenetic analyses. Bioinformatics 25, 1972–1973. doi: 10.1093/bioinformatics/btp348, PMID: 19505945 PMC2712344

[ref5] Fiołka M. J. Takeuchi N. Sofińska-Chmiel W. Wójcik-Mieszawska S. Irvine-Fynn T. Edwards A. (2021). Morphological and spectroscopic analysis of snow and glacier algae and their parasitic fungi on different glaciers of Svalbard. Sci. Rep. 11:21785. doi: 10.1038/s41598-021-01211-8, PMID: 34750421 PMC8575968

[ref6] Freeman K. R. Martin A. P. Karki D. Lynch R. C. Mitter M. S. Meyer A. F.. (2009). Evidence that chytrids dominate fungal communities in high-elevation soils. Proc. Natl. Acad. Sci. U. S. A. 106, 18315–18320. doi: 10.1073/pnas.0907303106 19826082 PMC2775327

[ref7] Frenken T. Alacid E. Berger S. A. Bourne E. C. Gerphagnon M. Grossart H.-P.. (2017). Integrating chytrid fungal parasites into plankton ecology: research gaps and needs. Environ. Microbiol. 19, 3802–3822. doi: 10.1111/1462-2920.13827, PMID: 28618196

[ref8] Ganey G. Q. Loso M. G. Burgess A. B. Dial R. J. (2017). The role of microbes in snowmelt and radiative forcing on an Alaskan icefield. Nat. Geosci. 10, 754–759. doi: 10.1038/ngeo3027

[ref9] Gardes M. Bruns T. D. (1993). ITS primers with enhanced specificity for basidiomycetes – application to the identification of mycorrhizae and rusts. Mol. Ecol. 2, 113–118. doi: 10.1111/j.1365-294X.1993.tb00005.x, PMID: 8180733

[ref10] Genitsaris S. Kormas K. A. Moustaka-Gouni M. (2009). Microscopic eukaryotes living in a dying Lake (Lake Koronia, Greece). FEMS Microbiol. Ecol. 69, 75–83. doi: 10.1111/j.1574-6941.2009.00686.x, PMID: 19453739

[ref11] Gromov B. V. Mamkaeva K. A. Pljusch A. V. (2000). Mesochytrium penetrans gen. et sp. nov. (Chytridiales) – a parasite of the green alga *Chlorococcum minutum* (Chlorococcales), with an unusual behaviour of the sporangia. Nova_Hedwigia 71, 151–160. doi: 10.1127/nova/71/2000/151

[ref12] Gsell A. S. De Senerpont Domis L. N. Naus-Wiezer S. M. H. Helmsing N. R. Van Donk E. Ibelings B. W. (2013). Spatiotemporal variation in the distribution of chytrid parasites in diatom host populations. Freshw. Biol. 58, 523–537. doi: 10.1111/j.1365-2427.2012.02786.x

[ref17] Hock R. Rasul, G. Adler C. Cáceres B. Gruber S. Hirabayashi Y.. (2019). “High Mountain Areas,” in IPCC Special Report on the Ocean and Cryosphere in a Changing Climate eds H. -O. Pörtner, D. C. Roberts, V. Masson-Delmotte, P. Zhai, M. Tignor, E. Poloczanska, et al. (Cambridge: Cambridge University Press), 131–202. doi: 10.1017/9781009157964.004

[ref14] Hoham R. W. Duval B. (2001). “Microbial ecology of snow and freshwater ice with emphasis on snow algae” in Snow ecology: an interdisciplinary examination of snow-covered ecosystems. eds. Jones H. G. Pomeroy J. W. Walker D. A. Hoham R. W. (Cambridge: Cambridge University Press), 168–288.

[ref15] Hoham R. W. Remias D. (2020). Snow and glacial algae: a review^1^. J. Phycol. 56, 264–282. doi: 10.1111/jpy.12952, PMID: 31825096 PMC7232433

[ref16] Hotaling S. Lutz S. Dial R. J. Anesio A. M. Benning L. G. Fountain A. G.. (2021). Biological albedo reduction on ice sheets, glaciers, and snowfields. Earth Sci. Rev. 220:103728. doi: 10.1016/j.earscirev.2021.103728

[ref18] Ishida S. Nozaki D. Grossart H.-P. Kagami M. (2015). Novel basal, fungal lineages from freshwater phytoplankton and lake samples. Environ. Microbiol. Rep. 7, 435–441. doi: 10.1111/1758-2229.12268, PMID: 25625632

[ref19] Jobard M. Rasconi S. Solinhac L. Cauchie H.-M. Sime-Ngando T. (2012). Molecular and morphological diversity of fungi and the associated functions in three European nearby lakes: true Fungi (Eumycota) in pelagic freshwaters. Environ. Microbiol. 14, 2480–2494. doi: 10.1111/j.1462-2920.2012.02771.x, PMID: 22568577

[ref20] Kagami M. de Bruin A. Ibelings B. W. Van Donk E. (2007). Parasitic chytrids: their effects on phytoplankton communities and food-web dynamics. Hydrobiologia 578, 113–129. doi: 10.1007/s10750-006-0438-z

[ref21] Kagami M. Seto K. Nozaki D. Nakamura T. Wakana H. Wurzbacher C. (2021). Single dominant diatom can host diverse parasitic fungi with different degree of host specificity. Limnol. Oceanogr. 66, 667–677. doi: 10.1002/lno.11631

[ref22] Kariya Y. (2005). Holocene landscape evolution of a nivation hollow on Gassan volcano, northern Japan. Catena 62, 57–76. doi: 10.1016/j.catena.2005.02.004

[ref23] Karpov S. A. Kobseva A. A. Mamkaeva M. A. Mamkaeva K. A. Mikhailov K. V. Mirzaeva G. S.. (2014). *Gromochytrium mamkaevae* gen. & sp. nov. and two new orders: Gromochytriales and Mesochytriales (Chytridiomycetes). Persoonia 32, 115–126. doi: 10.3767/003158514X680234, PMID: 25264386 PMC4150072

[ref24] Karpov S. A. Letcher P. M. Mamkaeva M. A. Mamkaeva K. A. (2010). Phylogenetic position of the genus *Mesochytrium* (Chytridiomycota) based on zoospore ultrastructure and sequences from the 18S and 28S rRNA gene. Nova_Hedwigia 90, 81–94. doi: 10.1127/0029-5035/2010/0090-0081

[ref25] Karpov S. A. Lуpez-Garcia P. Mamkaeva M. A. Vishnyakov A. E. Moreira D. (2016). Chytridiomycete *Polyphagus parasiticus*: molecular phylogeny supports the erection of a new chytridiomycete order. Mikologiya 50, 362–366.

[ref26] Karpov S. A. Moreira D. Mamkaeva M. A. Popova O. V. Aleoshin V. V. López-García P. (2019). New member of Gromochytriales (Chytridiomycetes)— *Apiochytrium granulosporum* nov. gen. et sp. J. Eukaryotic Microbiol. 66, 582–591. doi: 10.1111/jeu.12702, PMID: 30460733 PMC6685791

[ref27] Katoh K. Rozewicki J. Yamada K. D. (2019). MAFFT online service: multiple sequence alignment, interactive sequence choice and visualization. Brief. Bioinform. 20, 1160–1166. doi: 10.1093/bib/bbx108, PMID: 28968734 PMC6781576

[ref28] Kawase H. Yamazaki T. Sugimoto S. Sasai T. Ito R. Hamada T.. (2020). Changes in extremely heavy and light snow-cover winters due to global warming over high mountainous areas in Central Japan. Prog Earth Planet Sci 7:10. doi: 10.1186/s40645-020-0322-x

[ref29] Klawonn I. Dunker S. Kagami M. Grossart H.-P. Van den Wyngaert S. (2023). Intercomparison of two fluorescent dyes to visualize parasitic fungi (Chytridiomycota) on phytoplankton. Microb. Ecol. 85, 9–23. doi: 10.1007/s00248-021-01893-7 34854932 PMC9849195

[ref30] Kobayashi Y. Okubo M. (1954). “Studies on the aquatic fungi of the Ozegahara Moor,” in Scientific research of the Ozegahara Moor, ed. Scientific Researchers of the Ozegahara Moor, Japan Society for the Promotion of Science, 561–584.

[ref31] Kobayashi K. Takeuchi N. Kagami M. (2023). High prevalence of parasitic chytrids infection of glacier algae in cryoconite holes in Alaska. Sci. Rep. 13:3973. doi: 10.1038/s41598-023-30721-w, PMID: 36894609 PMC9998860

[ref32] Kol E. (1942). The snow and ice algae of Alaska. Smithsonian Misc. Collect. 101, 1–50.

[ref33] Kol E. (1968). “Kryobiologie. Biologie und Limnologie des Schneesund Eises” in Die Binnengewa¨sser, Band XXIV. ed. Kryovegetation I. (Stuttgart: Schweizerbart’sche Verlagsbuchhandlung)

[ref34] Krug L. Erlacher A. Markut K. Berg G. Cernava T. (2020). The microbiome of alpine snow algae shows a specific inter-kingdom connectivity and algae-bacteria interactions with supportive capacities. ISME J. 14, 2197–2210. doi: 10.1038/s41396-020-0677-4, PMID: 32424246 PMC7608445

[ref35] Lazarus K. L. James T. Y. (2015). Surveying the biodiversity of the Cryptomycota using a targeted PCR approach. Fungal Ecol. 14, 62–70. doi: 10.1016/j.funeco.2014.11.004

[ref36] Lefèvre E. Roussel B. Amblard C. Sime-Ngando T. (2008). The molecular diversity of freshwater picoeukaryotes reveals high occurrence of putative parasitoids in the plankton. PLoS One 3:e2324. doi: 10.1371/journal.pone.0002324, PMID: 18545660 PMC2396521

[ref37] Luo W. Kotut K. Krienitz L. (2013). Hidden diversity of eukaryotic plankton in the soda Lake Nakuru, Kenya, during a phase of low salinity revealed by a SSU rRNA gene clone library. Hydrobiologia 702, 95–103. doi: 10.1007/s10750-012-1310-y

[ref38] Lutz S. Anesio A. M. Raiswell R. Edwards A. Newton R. J. Gill F.. (2016). The biogeography of red snow microbiomes and their role in melting arctic glaciers. Nat. Commun. 7:11968. doi: 10.1038/ncomms11968, PMID: 27329445 PMC4917964

[ref39] Matsuzaki R. Kawai-Toyooka H. Hara Y. Nozaki H. (2015). Revisiting the taxonomic significance of aplanozygote morphologies of two cosmopolitan snow species of the genus *Chloromonas* (Volvocales, Chlorophyceae). Phycologia 54, 491–502. doi: 10.2216/15-33.1

[ref40] Matsuzaki R. Nozaki H. Kawachi M. (2018). Taxonomic revision of Chloromonas nivalis (Volvocales, Chlorophyceae) strains, with the new description of two snow-inhabiting *Chloromonas* species. PLoS One 13:e0193603. doi: 10.1371/journal.pone.0193603, PMID: 29570718 PMC5865719

[ref41] Matsuzaki R. Nozaki H. Takeuchi N. Hara Y. Kawachi M. (2019). Taxonomic re-examination of “*Chloromonas nivalis* (Volvocales, Chlorophyceae) zygotes” from Japan and description of *C. muramotoi* sp. nov. PLoS One 14:e0210986. doi: 10.1371/journal.pone.0210986, PMID: 30677063 PMC6345437

[ref43] Naff C. S. Darcy J. L. Schmidt S. K. (2013). Phylogeny and biogeography of an uncultured clade of snow chytrids. Environ. Microbiol. 15, 2672–2680. doi: 10.1111/1462-2920.12116, PMID: 23551529

[ref44] Nakashima T. Uetake J. Segawa T. Procházková L. Tsushima A. Takeuchi N. (2021). Spatial and temporal variations in pigment and species compositions of snow algae on Mt. Tateyama in Toyama prefecture, Japan. Front. Plant Sci. 12:689119. doi: 10.3389/fpls.2021.689119, PMID: 34290725 PMC8289405

[ref45] O’Donnell K. Cigelnik E. Benny G. L. (1998). Phylogenetic relationships among the Harpellales and Kickxellales. Mycologia 90, 624–639. doi: 10.1080/00275514.1998.12026952

[ref46] Ono M. Takeuchi N. Zawierucha K. (2021). Snow algae blooms are beneficial for microinvertebrates assemblages (Tardigrada and Rotifera) on seasonal snow patches in Japan. Sci. Rep. 11:5973. doi: 10.1038/s41598-021-85462-5, PMID: 33727649 PMC7971028

[ref47] Procházková L. Leya T. Křížková H. Nedbalová L. (2019). *Sanguina nivaloides* and *Sanguina aurantia* gen. et spp. nov. (Chlorophyta): the taxonomy, phylogeny, biogeography and ecology of two newly recognised algae causing red and orange snow. FEMS Microbiol. Ecol. 95:fiz064. doi: 10.1093/femsec/fiz064, PMID: 31074825 PMC6545352

[ref48] Raghavendra Rao N. N. Pavgi M. S. (1979). Germination of resting spores in *Synchytrium* species parasitic on cucurbitaceae. Mycopathologia 69, 3–10. doi: 10.1007/BF00428598

[ref13] R Core Team (2022). R: A language and environment for statistical computing. R Foundation for Statistical Computing, Vienna, Austria. Available at: https://www.R-project.org/

[ref49] Remias D. Karsten U. Lütz C. Leya T. (2010). Physiological and morphological processes in the alpine snow alga *Chloromonas nivalis* (Chlorophyceae) during cyst formation. Protoplasma 243, 73–86. doi: 10.1007/s00709-010-0123-y, PMID: 20229328

[ref50] Řezanka T. Nedbalová L. Sigler K. Cepák V. (2008). Identification of astaxanthin diglucoside diesters from snow alga *Chlamydomonas nivalis* by liquid chromatography–atmospheric pressure chemical ionization mass spectrometry. Phytochemistry 69, 479–490. doi: 10.1016/j.phytochem.2007.06.025, PMID: 17681561

[ref51] Schmidt S. K. Naff C. S. Lynch R. C. (2012). Fungal communities at the edge: ecological lessons from high alpine fungi. Fungal Ecol. 5, 443–452. doi: 10.1016/j.funeco.2011.10.005

[ref52] Segawa T. Matsuzaki R. Takeuchi N. Akiyoshi A. Navarro F. Sugiyama S.. (2018). Bipolar dispersal of red-snow algae. Nat. Commun. 9:3094. doi: 10.1038/s41467-018-05521-w, PMID: 30082897 PMC6079020

[ref53] Seto K. Kagami M. Degawa Y. (2017). Phylogenetic position of parasitic chytrids on diatoms: characterization of a novel clade in chytridiomycota. J. Eukaryot. Microbiol. 64, 383–393. doi: 10.1111/jeu.12373, PMID: 27714973

[ref54] Simmons D. R. James T. Y. Meyer A. F. Longcore J. E. (2009). Lobulomycetales, a new order in the Chytridiomycota. Mycol. Res. 113, 450–460. doi: 10.1016/j.mycres.2008.11.019, PMID: 19138737

[ref55] Takeuchi N. (2001). The altitudinal distribution of snow algae on an Alaska glacier (Gulkana Glacier in the Alaska Range). Hydrol. Process. 15, 3447–3459. doi: 10.1002/hyp.1040

[ref56] Terashima M. Umezawa K. Mori S. Kojima H. Fukui M. (2017). Microbial community analysis of colored snow from an alpine snowfield in Northern Japan reveals the prevalence of Betaproteobacteria with snow algae. Front. Microbiol. 8:1481. doi: 10.3389/fmicb.2017.01481, PMID: 28824603 PMC5545588

[ref57] Thomas M. C. Selinger L. B. Inglis G. D. (2012). Seasonal diversity of planktonic protists in Southwestern Alberta Rivers over a 1-year period as revealed by terminal restriction fragment length polymorphism and 18S rRNA gene library analyses. Appl. Environ. Microbiol. 78, 5653–5660. doi: 10.1128/AEM.00237-12, PMID: 22685143 PMC3406148

[ref58] Trifinopoulos J. Nguyen L. T. von Haeseler A. Minh B. Q. (2016). W-IQ-TREE: a fast online phylogenetic tool for maximum likelihood analysis. Nucleic Acids Res. 44, W232–W235. doi: 10.1093/nar/gkw256, PMID: 27084950 PMC4987875

[ref59] Truett G. E. Heeger P. Mynatt R. L. Truett A. A. Walker J. A. Warman M. L. (2000). Preparation of PCR-quality mouse genomic DNA with hot sodium hydroxide and tris (HotSHOT). BioTechniques 29, 52–54. doi: 10.2144/00291bm09, PMID: 10907076

[ref60] Van den Wyngaert S. Ganzert L. Seto K. Rojas-Jimenez K. Agha R. Berger S. A.. (2022). Seasonality of parasitic and saprotrophic zoosporic fungi: linking sequence data to ecological traits. ISME J. 16, 2242–2254. doi: 10.1038/s41396-022-01267-y, PMID: 35764676 PMC9381765

[ref61] Van den Wyngaert S. Rojas-Jimenez K. Seto K. Kagami M. Grossart H.-P. (2018). Diversity and hidden host specificity of chytrids infecting colonial volvocacean algae. J. Eukaryot. Microbiol. 65, 870–881. doi: 10.1111/jeu.12632, PMID: 29752884

[ref4] Van Donk E. Ringelberg J. (1983). The effect of fungal parasitism on the succession of diatoms in Lake Maarsseveen I (The Netherlands). Freshw. Biol. 13, 241–251. doi: 10.1111/j.1365-2427.1983.tb00674.x

[ref62] Vilgalys R. Hester M. (1990). Rapid genetic identification and mapping of enzymatically amplified ribosomal DNA from several *Cryptococcus* species. J. Bacteriol. 172, 4238–4246. doi: 10.1128/jb.172.8.4238-4246.1990, PMID: 2376561 PMC213247

[ref63] White T. J. Bruns T. Lee S. Taylor J. (1990). “Amplification and direct sequencing of fungal ribosomal RNA genes for phylogenetics” in PCR protocols: a guide to methods and applications. eds. Innis M. A. Gelfand D. H. Sninsky J. J. White T. J. (New York: Academic Press), 315–322.

[ref64] Wurzbacher C. Larsson E. Bengtsson-Palme J. van den Wyngaert S. Svantesson S. Kristiansson E.. (2019). Introducing ribosomal tandem repeat barcoding for fungi. Mol. Ecol. Resour. 19, 118–127. doi: 10.1111/1755-0998.12944, PMID: 30240145

[ref65] Yakimovich K. M. Engstrom C. B. Quarmby L. M. (2020). Alpine snow algae microbiome diversity in the coast range of British Columbia. Front. Microbiol. 11:1721. doi: 10.3389/fmicb.2020.01721, PMID: 33013720 PMC7485462

