# Supplementary material for: Novel parasitic chytrids infecting snow algae in an alpine snow ecosystem in Japan
Source: Front Microbiol. 2023 Jun 20;14:1201230. doi: 10.3389/fmicb.2023.1201230 (PMC10318532; doi:10.3389/fmicb.2023.1201230)
Supplement: Supplementary file 1 [file Table_1.DOCX]

Supplementary Material

Novel parasitic chytrids infecting snow algae in an alpine snow ecosystem in Japan

Hiroaki Nakanishi^*^, Kensuke Seto^2^, Nozomu Takeuchi^3^, Maiko Kagami^*^

*** Correspondence:** Hiroaki Nakanishi and Maiko Kagami: [nakanishi-hiroaki-py@ynu.jp](mailto:nakanishi-hiroaki-py@ynu.jp) and [kagami-maiko-bd@ynu.ac.jp](mailto:kagami-maiko-bd@ynu.ac.jp)

# Supplementary Table

**Table S1.** List of isolated cells of parasitic chytrids infecting *Chloromonas* spp. and the success or failure of PCR amplification and sequencing. Circles indicate successful sequencing.

| **Sample name** | **Storage condition** | **Morphological type** | **18S** | | **ITS1-5.8S-ITS2** | | **28S** | |
| --- | --- | --- | --- | --- | --- | --- | --- | --- |
|  |  |  | **PCR amplification** | **Sequence** | **PCR amplification** | **Sequence** | **PCR amplification** | **Sequence** |
| 21GAbe-1 | Incubation | Unclassified | ○ | － | － | － | ○ | － |
| 21GAbe-2 | Incubation | Unclassified | － | － | － | － | － | － |
| 21GAbe-3 | Incubation | Resting spore | － | － | － | － | － | － |
| 21GAbe-4 | Incubation | Type 1 | ○ | ○ | － | － | ○ | － |
| 21GAbe-5 | Incubation | Resting spore | ○ | － | － | － | ○ | － |
| 21GAbe-6 | Incubation | Resting spore | － | － | － | － | － | － |
| 21GAbe-7 | Incubation | Type 2 | ○ | ○ | － | － | ○ | － |
| 21GAbe-8 | Incubation | Unclassified | － | － | － | － | － | － |
| 21GAbe-9 | Incubation | Unclassified | － | － | － | － | － | － |
| 21GAbe-10 | Incubation | Type 1 | － | － | － | － | － | － |
| 21GAbe-11 | Incubation | Type 1 | － | － | － | － | － | － |
| 21GAbe-12 | Incubation | Unclassified | － | － | － | － | － | － |
| 21GAbe-13 | Incubation | Resting spore | － | － | － | － | － | － |
| 21GAbe-14 | Incubation | Resting spore | － | － | － | － | － | － |
| 21GAbe-15 | Incubation | Type 2 | － | － | － | － | － | － |
| 21GAbe-16 | Incubation | Resting spore | － | － | － | － | － | － |
| 21GAbe-17 | Incubation | Resting spore | － | － | － | － | － | － |
| 21GAbe-18 | Incubation | Resting spore | － | － | － | － | － | － |
| 21GAbe-19 | Incubation | Type 2 | － | － | － | － | － | － |
| 21GAbe-20 | Incubation | Resting spore | － | － | － | － | － | － |
| 21GAbe-21 | Incubation | Type 2 | － | － | － | － | － | － |
| 21GAbe-22 | Incubation | Resting spore | － | － | － | － | － | － |
| 21GAbe-23 | Incubation | Resting spore | － | － | － | － | － | － |
| 21GAbe-24 | Incubation | Type 1 | － | － | － | － | － | － |
| 21GAbe-25 | Incubation | Type 1 | － | － | － | － | － | － |
| 21GAbe-26 | Incubation | Resting spore | － | － | － | － | － | － |
| 21GAbe-27 | Incubation | Type 2 | － | － | － | － | － | － |
| 21GAbe-28 | Incubation | Resting spore | － | － | － | － | － | － |
| 21GAbe-29 | Incubation | Type 2 | － | － | － | － | － | － |
| 21GAbe-30 | Incubation | Resting spore | － | － | － | － | － | － |
| 21GAbe-31 | Incubation | Resting spore | － | － | － | － | － | － |
| 21GAbe-32 | Incubation | Resting spore | － | － | － | － | － | － |
| 21GAbe-33 | Incubation | Resting spore | － | － | － | － | － | － |
| 21GAbe-34 | Incubation | Resting spore | － | － | － | － | － | － |
| 21GAbe-35 | Incubation | Resting spore | － | － | － | － | － | － |
| 21GAbe-36 | Incubation | Resting spore | － | － | － | － | － | － |
| 21GAbe-37 | Incubation | Resting spore | － | － | － | － | － | － |
| 21GAbe-38 | Incubation | Resting spore | － | － | － | － | － | － |
| 21GAbe-39 | Cryopreserved | Type 1 | － | － | － | － | － | － |
| 21GAbe-40 | Cryopreserved | Type 1 | － | － | － | － | － | － |
| 21GAbe-41 | Cryopreserved | Type 1 | － | － | － | － | － | － |
| 21GAbe-42 | Cryopreserved | Type 1 | ○ | ○ | ○ | ○ | ○ | ○ |
| 21GAbe-43 | Cryopreserved | Type 1 | ○ | ○ | ○ | ○ | ○ | ○ |
| 21GAbe-44 | Cryopreserved | Type 1 | ○ | ○ | ○ | ○ | ○ | ○ |
| 21GAsk-1 | Cryopreserved | Type 3 | ○ | ○ | ○ | ○ | ○ | ○ |
| 21GAsk-2 | Cryopreserved | Type 3 | － | － | ○ | ○ | ○ | ○ |
| 21GAsk-3 | Cryopreserved | Type 3 | ○ | ○ | ○ | ○ | ○ | ○ |
| 21GAsk-4 | Cryopreserved | Type 3 | － | － | ○ | ○ | ○ | ○ |
| 22GAbe-1 | Cryopreserved | Type 2 | － | － | ○ | ○ | － | － |
| 22GAbe-2 | Cryopreserved | Type 2 | － | － | ○ | ○ | － | － |
| 22GAbe-3 | Cryopreserved | Type 2 | － | － | － | － | － | － |
| 22GAbe-4 | Cryopreserved | Type 2 | ○ | ○ | ○ | ○ | ○ | ○ |
| 22GAbe-5 | Cryopreserved | Type 2 | ○ | ○ | ○ | ○ | ○ | ○ |
| 22GAbe-6 | Cryopreserved | Type 2 | － | － | ○ | ○ | － | － |
| 22GAbe-7 | Cryopreserved | Type 2 | － | － | ○ | ○ | － | － |
| 22GAbe-8 | Cryopreserved | Type 2 | － | － | ○ | ○ | ○ | ○ |
| 22GAbe-9 | Cryopreserved | Type 2 | － | － | ○ | ○ | － | － |
| 22GAbe-10 | Cryopreserved | Type 2 | － | － | ○ | ○ | － | － |
| 22GAbe-11 | Cryopreserved | Type 2 | － | － | － | － | － | － |
| 22GAbe-12 | Cryopreserved | Type 2 | ○ | ○ | ○ | ○ | － | － |
| 22GAbe-13 | Cryopreserved | Type 2 | － | － | － | － | － | － |
| 22GAbe-14 | Cryopreserved | Type 2 | － | － | ○ | ○ | － | － |
| 22GAbe-15 | Cryopreserved | Type 2 | － | － | ○ | ○ | － | － |
| 22GAbe-16 | Cryopreserved | Type 2 | － | － | ○ | ○ | ○ | ○ |
| 22GAbe-17 | Cryopreserved | Type 2 | － | － | ○ | ○ | － | － |
| 22GAbe-18 | Cryopreserved | Type 2 | － | － | ○ | ○ | ○ | ○ |
| 22GAbe-19 | Cryopreserved | Type 2 | － | － | ○ | ○ | ○ | ○ |
| 22GAbe-20 | Cryopreserved | Type 2 | － | － | ○ | ○ | － | － |
| 22GAbe-21 | Cryopreserved | Type 2 | － | － | ○ | ○ | － | － |
| 22GAbe-22 | Cryopreserved | Type 2 | － | － | － | － | － | － |
| 22GAbe-23 | Cryopreserved | Type 2 | － | － | － | － | － | － |
| 22GAbe-24 | Cryopreserved | Type 2 | － | － | ○ | ○ | － | － |
| 22GAbe-25 | Cryopreserved | Type 2 | － | － | ○ | ○ | － | － |
| 22GAbe-26 | Cryopreserved | Type 2 | － | － | － | － | ○ | ○ |
| 22GAbe-27 | Cryopreserved | Type 2 | － | － | － | － | － | － |
| 22GAbe-28 | Cryopreserved | Type 2 | － | － | － | － | － | － |
| 22GAbe-29 | Cryopreserved | Type 2 | ○ | ○ | ○ | ○ | ○ | ○ |
| 22GAbe-30 | Cryopreserved | Type 2 | － | － | ○ | ○ | ○ | ○ |
| 22GAbe-31 | Cryopreserved | Type 2 | － | － | ○ | ○ | － | － |
